# High-Resolution Translatome Analysis Reveals Cortical Cell Programs During Early Soybean Nodulation

**DOI:** 10.3389/fpls.2022.820348

**Published:** 2022-04-14

**Authors:** Jae Hyo Song, Bruna Montes-Luz, Michelle Zibetti Tadra-Sfeir, Yaya Cui, Lingtao Su, Dong Xu, Gary Stacey

**Affiliations:** ^1^Divisions of Plant Sciences and Biochemistry, Christopher S. Bond Life Sciences Center, University of Missouri, Columbia, MO, United States; ^2^Department of Electrical Engineering and Computer Science, C.S. Bond Life Science Center, University of Missouri, Columbia, MO, United States

**Keywords:** TRAP-seq, cortical cell, soybean, nodulation, phytohormone

## Abstract

Nodule organogenesis in legumes is regulated temporally and spatially through gene networks. Genome-wide transcriptome, proteomic, and metabolomic analyses have been used previously to define the functional role of various plant genes in the nodulation process. However, while significant progress has been made, most of these studies have suffered from tissue dilution since only a few cells/root regions respond to rhizobial infection, with much of the root non-responsive. To partially overcome this issue, we adopted translating ribosome affinity purification (TRAP) to specifically monitor the response of the root cortex to rhizobial inoculation using a cortex-specific promoter. While previous studies have largely focused on the plant response within the root epidermis (e.g., root hairs) or within developing nodules, much less is known about the early responses within the root cortex, such as in relation to the development of the nodule primordium or growth of the infection thread. We focused on identifying genes specifically regulated during early nodule organogenesis using roots inoculated with *Bradyrhizobium japonicum*. A number of novel nodulation gene candidates were discovered, as well as soybean orthologs of nodulation genes previously reported in other legumes. The differential cortex expression of several genes was confirmed using a promoter-GUS analysis, and RNAi was used to investigate gene function. Notably, a number of differentially regulated genes involved in phytohormone signaling, including auxin, cytokinin, and gibberellic acid (GA), were also discovered, providing deep insight into phytohormone signaling during early nodule development.

## Introduction

Legumes have a unique impact on the nitrogen cycle and play an important role in agriculture and natural ecosystems. They normally do so by establishing a symbiotic relationship with soil rhizobia, nitrogen-fixing soil bacteria. These bacteria infect legume roots giving rise to a novel organ, the nodule, which they colonize, providing fixed nitrogen for plant growth (Ferguson et al., 2010). This allows legumes to grow without the addition of nitrogen fertilizers, contributing to a better energy balance and more sustainable crop production.

The establishment of an intimate symbiosis between rhizobia and host plants requires the exchange of diffusible chemical signals. Flavonoids are released by the host plants in root exudates to attract rhizobia and induce gene expression (Shultz et al., 2006; Sugiyama et al., 2008). Induction of the rhizobial nodulation genes results in the synthesis of the lipochitooligosaccharide Nod factors (NFs), which are recognized by the plant and are responsible for inducing key events in the infection process (Stacey et al., 2006; Oldroyd, 2013; Liang et al., 2014). When NFs are recognized by NF receptors, legumes initiate a series of biochemical cascades, including regular calcium oscillations in and around the nuclei of root epidermal cells (Oldroyd and Downie, 2008), which activate downstream gene expression of nodulation-specific genes, such as *NODULATION-SIGNALING PATHWAY 1* (*NSP1*), *NSP2*, *Nodule Inception* (*NIN*), and *ENOD40* (Fang and Hirsch, 1998; Schauser et al., 1999; Heckmann et al., 2006; Oldroyd and Downie, 2008; Vernié et al., 2008). The plant hormone cytokinin is the primary hormone needed for nodule organogenesis (Miri et al., 2016; Gamas et al., 2017). Additional plant hormones act to either promote nodulation (e.g., auxin, brassinosteroids, and gibberilins) or inhibit nodulation [e.g., jasmonic acid (JA) or ethylene] (Mathews et al., 1989; Ferguson and Mathesius, 2003; Ferguson et al., 2005; Sun et al., 2006; Kinkema and Gresshoff, 2008).

In the past few decades, forward genetic screens have identified a number of plant genes that are required for efficient nodule formation and nitrogen fixation (Roy et al., 2020). In addition to these studies, a number of genome-wide transcriptomic, proteomic, and metabolomic studies have also been used to profile legume nodulation, with many focused on early events in the infection process (Libault et al., 2010; Brechenmacher et al., 2012; Nguyen et al., 2012). A limitation in most of these studies is that only a small region of the legume root is responsive to rhizobial infection with most of the root non-responsive. Hence, studies sampling whole roots suffer from tissue dilution where transcripts/proteins/metabolites from non-responding tissues can obscure changes occurring in response to rhizobial inoculation. In the past studies, we pointed to these problems and sought to reduce their impact by specifically sampling soybean root hair cells, which are the primary site of rhizobial infection (Libault et al., 2010). However, even in this case, only a few root hair cells respond to inoculation with most showing no response.

**FIGURE 1:**
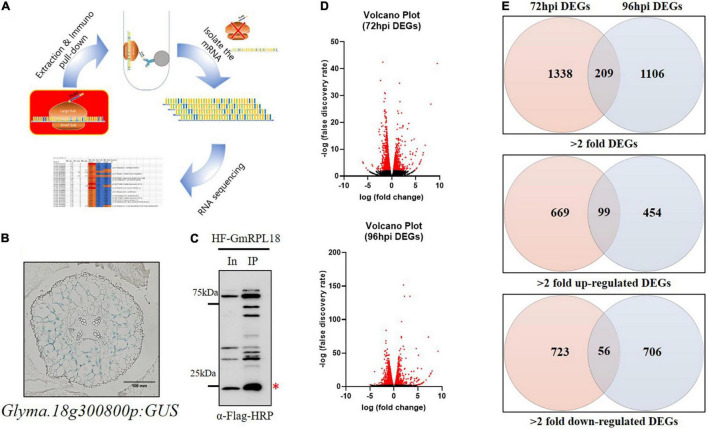
Analysis of genome-wide expressional changes in response to *Bj* infection in the cortex layer using TRAP-seq. **(A)** Principles of the TRAP method. Transgenic hairy roots expressing HF-GmRPL18 protein were used for further protein extraction and immunoprecipitation, followed by mRNA isolation and sequencing. **(B)** Cortex-specific expression of *Glyma.18g300800* in uninoculated soybean transgenic hairy root expressing a promoter-GUS construct. **(C)** Western blot analysis with total and immunoprecipitated proteins. Immuno-pulldown was performed using Anti-FLAG conjugated bead and then protein was detected by anti-FLAG-HRP (Red asterisk indicates the HF-GmRPL18 protein, In: Input protein, IP: Immunoprecipitated protein) **(D)** volcano plots represent DEGs with significant expressional changes (red) separated by expressional change (log2FC) vs. false discovery rate (-log10). **(E)** Venn diagrams of up-regulated or down-regulated DEGs (Cutoff: ≥ 2 = fold change for each up-regulated and down-regulated DEGs; *p* ≤ 0.05).

Plant genomes can encode tens of thousands of genes, but many are expressed in only a few organs, tissues, or cell types. It is a real challenge to measure gene expression in a particular cell type (Rogers et al., 2012). Recently, fluorescence-activated cell sorting (FACS) of protoplasts or laser capture microdissection (LCM) have been used to gain insight into genome-wide expression at the cell-layer level (Birnbaum et al., 2005; Celedon et al., 2017). In addition, TRAP-seq [translating ribosome affinity purification (TRAP) followed by RNA sequencing] allows for the investigation of genome-wide expression changes at the cell-layer level using promoters that show cell-specific expression (Mustroph et al., 2009; Sorenson and Bailey-Serres, 2015). This method uses cell type-specific expression of FLAG-tagged ribosomes that allows subsequent isolation of ribosome-mRNA complexes by co-immunoprecipitation and subsequent RNA sequencing to obtain expression profiles. These data are referred to as cell layer-specific translatomes. Our laboratory and collaborators successfully adapted this technology to soybean using a FLAG-tagged soybean RPL18 subunit (*Glyma20g38130*) (Castro-Guerrero et al., 2016). In addition, using LCM followed by transcriptomic analysis, we identified a highly cortex-specific promoter (*Glyma.18g53890*, Figure 1B). To identify genes involved in early nodule organogenesis and infection thread progression, we combined the aforementioned cortex-specific TRAP followed by RNAseq to identify cortex-specific genes differentially expressed upon *B. japonicum* inoculation. This enabled us to investigate the early nodulation process at a very high resolution. Additionally, we were able to assess gene expression more precisely by identifying numerous well-characterized genes involved in nodule development within cortical cells. By specifically targeting a stage of the nodulation process where we have relatively little understanding (e.g., cortex-specific responses), the data obtained expand our knowledge and provide a toolset of genes that can be used to further our understanding of the rhizobial infection process.

## Results

### Translating Ribosome Affinity Purification Is Effective in Isolating Cortex-Specific mRNA

The TRAP-seq process is dependent on the expression of a cell layer-specific His-FLAG-tagged ribosomal protein L18 (HF-GmRPL18), which allows for the immunoprecipitation of ribosomes with their corresponding mRNA to produce tissue-specific translatomes (Figure 1A; Zanetti et al., 2005; Castro-Guerrero et al., 2016).

To study rhizobial-induced transcriptional changes in the cortex during early nodule development, we identified a soybean promoter (*Glyma.18g300800*, Figure 1B) expressed exclusively in the cortex cells using LCM (Kerk et al., 2003; Casson et al., 2008) followed by transcriptional analysis. The cortex-specific promoter was used to drive the expression of GmRPL18 in soybean hairy roots. To capture events occurring in the cortex during the early stages of infection and initial cortical cell divisions, we inoculated plants and performed a time-course collection of root samples at 72 and 96-h post inoculation (hpi) followed by TRAP-seq. Immunoblot analysis indicated that an adequate amount of protein was present for immunoprecipitation (Figure 1C). Taken together, time-course cortex-specific TRAP-seq was established for studying early nodule development in soybean.

### Translating Ribosome Affinity Purification-Sequencing Allowed for the Identification of Differentially Expressed Genes in the Cortical Cell Layer

To profile the transcriptional changes associated with cortex-specific soybean genes upon *B. japonicum* inoculation, we performed RNA-seq on the mRNA isolated with TRAP using the Illumina NextSeq 500. Three biological replicates of root samples were harvested at each time point, either with or without *B. japonicum* inoculation. At least 11 million total reads and a minimum of 14.5 percent to a maximum of 30.8 percent unmapped reads were generated using 75-bp paired-end reads (Table 1). Using this stringent filtering method, each of the reads aligned to the annotated soybean genome (available at Phytozome).^1^ We determined differentially expressed genes (DEGs) in 72 and 96 hpi samples using edgeR (Robinson et al., 2009), as shown in the volcano plots (Figure 1D). As a result, 1,547 and 1,315 significant DEGs (>2-fold) were generated in the 72 and 96 hpi samples, respectively.

**TABLE 1:** Illumina RNA-seq output and mapping of 75-bp reads.

Sample	# of total reads	# of unmapped reads	% of unmapped reads	# of mapped reads	% of mapped reads	# of multimapping reads
72 h mock-1	15,679,114	2,345,455	15.0	13,333,659	85.0	1,209,489
72 h mock-2	16,103,859	2,342,754	14.5	13,761,105	85.5	1,241,998
72 h mock-3	15,049,546	2,209,316	14.7	12,840,230	85.3	1,154,613
72 h *Bj*-1	12,185,783	2,217,864	18.2	9,967,919	81.8	928,766
72 h *Bj*-2	12,662,726	2,392,181	18.9	10,270,545	81.1	999,423
72 h *Bj*-3	11,731,925	2,108,800	18.0	9,623,125	82.0	980,344
96 h mock-1	24,739,047	5,849,271	23.6	18,889,776	76.4	1,825,215
96 h mock-2	29,150,782	6,938,726	23.8	22,212,056	76.2	2,167,649
96 h mock-3	28,312,046	6,888,229	24.3	21,423,817	75.7	2,094,948
96 h *Bj*-1	27,869,985	8,576,902	30.8	19,293,083	69.2	1,910,146
96 h *Bj*-2	25,073,752	6,493,595	25.9	18,580,157	74.1	1,800,428
96 h *Bj*-3	27,553,167	7,838,225	28.4	19,714,942	71.6	1,932,660

Temporal and spatial gene regulation is critical during soybean nodulation signaling. Interestingly, roughly 20 and 10% of genes were differentially expressed in the 72 and 96 hpi samples in both up- and down-regulated DEGs, respectively (Figure 1E and Supplementary Table 1). This result is consistent with the view that early nodulation events are controlled by dynamic temporal gene regulation. Around 100 DEGs were upregulated in both time points following *B. japonicum* inoculation, suggesting a set of cortex-specific nodulation regulated genes.

**FIGURE 2:**
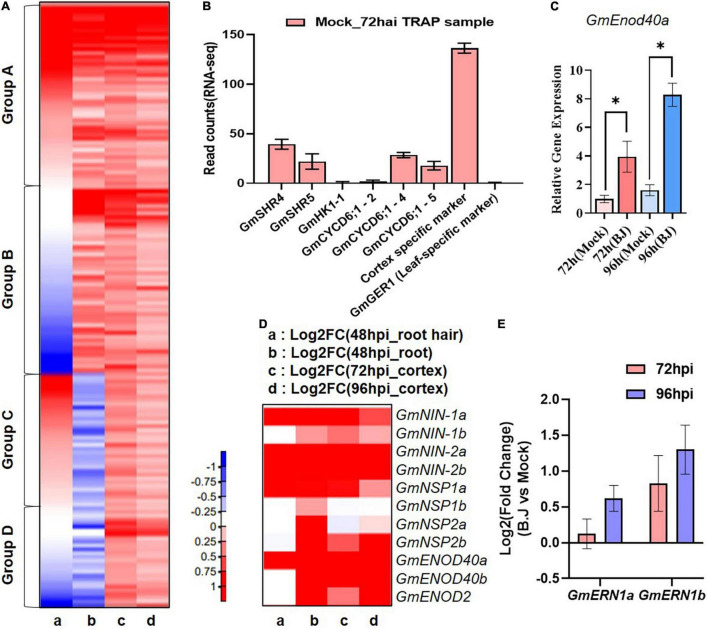
Comparison of gene expression during early nodule development. **(A)** Heatmap of RNA-seq transcriptome analysis for 640 selected genes in (a) 48 hpi_root hair (Libault et al., 2010) (b) 48 hpi_root (Hayashi et al., 2012) (c) 72 hpi_TRAP-seq (d) 96 hpi_TRAP-seq. Heatmap were generated base on Log2 fold change of gene expression in response to *B. japonicum* inoculation ranging from decreased (blue) to increased (red) as show in the color gradient at the bottom right corner. **(B)** Expression of the vascular-specific genes in TRAP-seq data. **(C)** q-RT PCR of *GmEnod40b* in the corresponding whole transgenic hairy root samples with or without *B. japonicum* (Black asterisk indicate the *t*-test significance at *p* < 0.001). **(D)** Heatmap of well-characterized nodule-associated genes in RNA-seq data sets. Heatmap were generated in the same way as **(A)**. **(E)** The expression of well-characterized nodule development-associated genes in the TRAP-seq data sets.

To emphasize the specificity of our TRAP-seq data, we examined the expression of cell type specific genes in our TRAP-seq data. For this experiment, we selected several soybean genes (*GmSHR4*, *GmSHR5*, *GmHK1-1*, *GmCYCD6;1-2*, *GmCYCD6;1-4*, and *GmCYCD6;1-5*) expressed in vascular tissue without *B. japonicum* treatment and previously confirmed by RNA *in situ* hybridization (Wang et al., 2022). The expression of these genes was evaluated using the cortex-specific marker used in our TRAP-seq experiment as a positive control and the leaf specific gene *GmGER1* as a negative control (Xun et al., 2021). In this study, we discovered that the expression levels *of GmHK1-1* and *GmCYCD6;1-2* were similar to *GmGER1* and other vascular tissue-specific genes (*GmSHR4*, *GmSHR5*, and *GmCYCD6;1-4*) showed low levels of expression when compared to the positive control in the TRAP-seq data (Figure 2B).

To characterize our TRAP-seq data, we examined cortex-expressed genes induced by *B. japonicum* inoculation. For this analysis, we compared 640 genes whose expression significantly (*p* < 0.05) increased in the 72 and 96 hai TRAP-seq data to previously reported root hair and root specific RNA-seq data (Libault et al., 2010; Hayashi et al., 2012). This analysis allowed classification of the genes into four groups relative to their expression patterns (Figure 2A and Supplementary Table 2). A total of 196 genes (Group A) were induced by rhizobia inoculation in all three RNA-seq data sets, whereas 196 genes (group B) were induced in the cortex and root-specific RNA-seq data, but not in root hairs, while 152 genes (group C) were induced in the cortex and root hair RNA-seq dataset but not in the root-specific data. Finally, 96 genes (Group D) were induced only in the cortex TRAP-seq data. According to this result, Group D represent novel candidate genes induced by rhizobia in the cortex that were not identified in previous soybean RNA-seq analyses. In addition, among the identified induced genes from the previous RNA-seq data, we could specify genes (Group B) that are likely to be induced in the cortex.

### Translating Ribosome Affinity Purification-Sequencing Allows for the Identification of Tissue-Specific Expression of Known Early Nodulation Genes

Prior to our TRAP experiment, we analyzed the expression of *GmEnod40b* as a marker gene for the cortex-specific nodulation responses and showed that this gene was strongly induced both at 72 and 96 h after inoculation (Figure 2C). To further confirm that our TRAP-seq data accurately profiled early soybean nodulation responses, we also examined the data for a variety of known nodulation genes. Among these were *GmENOD40a*, *GmENOD40b* (Kouchi and Hata, 1993), *NIN* (Wang et al., 2019), *NSP1* (Smit et al., 2005), and *NSP2* (Kaló et al., 2005).

In a previous study, four soybean orthologs (*GmNIN1a*, *GmNIN1b*, *GmNIN2a*, and *GmNIN2b*) of *NIN* were identified and shown to be up-regulated in a study that compared the plant response to inoculation with wild-type *B. japonicum* relative to inoculation with a Nod factor defective *B. japonicum* nodC mutant (Hayashi et al., 2012). In our TRAP-seq, these *NIN* orthologous genes were up-regulated in response to inoculation of *B. japonicum*. In the case of *GmNIN1b*, however, there was no significant difference in root hair RNA-seq data compare to the other three orthologs. Furthermore, *GmENOD40b* showed a similar expression pattern to that of *GmNIN1b*, both induced by *B. japonicum* inoculation, as previously shown in Hayashi et al. (2012) (Figure 2D and Supplementary Table 3). A similar situation was seen in *GmENOD40* ortholog genes. *GmENOD40a* was induced in the 3 RNA-seq data sets, while *GmENOD40b* was not induced in root hair RNA-seq data but was in the total root and cortex data sets. These results suggest that *GmNINb* and *GmEnod40b* likely play a role in nodulation events within the cortex, while their orthologs likely function in earlier nodulation events (Figure 2D and Supplementary Table 3).

In the case of *NSP* orthologs, *GmNSP1* and *GmNSP2* genes were up-regulated in response to *B. japonicum* in the previous total root RNA-seq data (Hayashi et al., 2012) but not all *NSP* genes were up-regulated in the root hair RNA-seq and our TRAP-seq data sets. *GmNSP1a* and *GmNSP2b* were up-regulated in inoculated samples while no difference in expression was detected for *GmNSP1b* and *GmNSP2a* in our TRAP-seq data and only *GmNSP1a* was induced in the root hair RNA-seq data set (Figure 2D and Supplementary Table 3). *NSP*s have been shown to regulate legume nodulation and to be induced by *B. japonicum* inoculation (Kaló et al., 2005; Hossain et al., 2019), but the spatiotemporal pattern of *NSP*s orthologs in the RNA-seq data sets was different (Figure 2D and Supplementary Table 3). Taken together, our findings suggest that *GmNSP1a* and *GmNSP2b* are likely important regulators of early nodule formation within the cortex layer, whereas *GmNSP1b* and *GmNSP2a* are not. As demonstrated in this example, we were able to accurately profile the soybean homologs in a number of previously studied legume nodule-related genes.

### Functional Analysis Uncovers Many Differentially Expressed Genes in the Root Cortex

To show the functional diversity of cortex-expressed genes induced by *B. japonicum* inoculation, we examined the functional classification of the 72 and 96 hpi-DEGs using the web-based MapMan annotation tool (Schwacke et al., 2019).^2^ The number of total genes belonging in each category was similar for the 72 and 96 hpi-DEGs (Supplementary Figure 1A). The largest category was Enzyme. RNA biosynthesis, solute transport, and phytohormones were sequentially annotated into the next larger categories (Supplementary Figure 1A and Supplementary Table 4).

We classified these genes further into sub-categories for each function. Numerous transcriptional regulators were classified into subgroups associated with RNA biosynthesis. The majority were found in the bHLH transcription factor subgroup, followed by the *MADS/AGL*, *C2H2-ZF*, *APETALA2/ETHYLENE RESPONSIVE FACTOR* (*AP2/ERF*), and *MYB*-related transcription factor subgroups (Supplementary Figure 1B and Supplementary Table 4). Within this subgroup of transcriptional regulators, *AP2/ERF* transcription factors have been implicated in the regulation of a variety of abiotic stresses and are hormone responsive (Dietz et al., 2010; Mizoi et al., 2012; Chandler, 2018). MADS-box transcription factors widely regulate most aspects of plant growth including the development of floral organs (Ma and Depamphilis, 2000). This subgroup analysis of transcription factors could serve as a starting point for discovering transcription factors that regulate early nodule development in the cortex layer. For instance, *ERN* is a transcription regulator that belongs to the *AP2/ERF* family and is known to be required for nodule formation, along with *NSP1* and *NSP2*. Both *ERN* homologs, *GmERN1a* and *GmERN1b*, were up-regulated in our data sets, but *GmERN1b* increased more markedly (Figure 2E). This result suggests that *GmERN1b* might be more important for nodule development than *GmERN1a* in the cortex layer.

Auxin and cytokinin seemed to be significant signaling molecules based on our analyses. Additionally, three auxin transporter genes were identified in the sub-group of solute transporters (Supplementary Figure 1C and Supplementary Table 4). These results support the hypothesis that auxin and cytokinin are the primary phytohormones involved in cortical nodule formation during the early stages of nodule formation. Taken together, this subgroup categorization contributes to our understanding of how phytohormones and their downstream genes regulate early nodule processes at the cortex cell layer.

### Candidate Genes Reveal Distinct Expression Profiles

**FIGURE 3:**
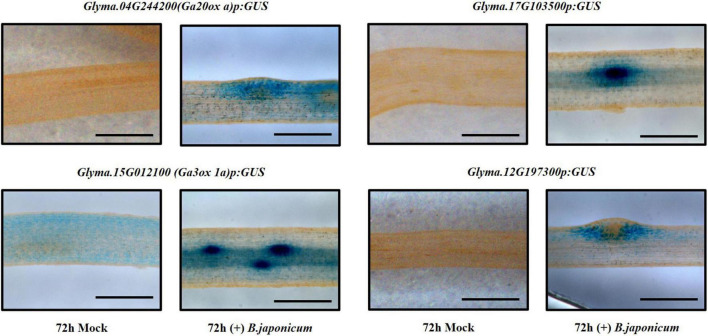
Promoter-GUS expression analysis of candidate genes, *Glyma.04G244200*, *Glyma.15G012100*, *Glyma.17G103500*, and *Glyma.12G197300* in soybean roots 72 h after inoculation with *B. japonicum* (scale bar = 500 μm).

To validate the expression of up-regulated genes in the TRAP-seq data set, we used promoter-GUS to analyze several candidates. Transgenic hairy root samples were collected 72 and 96 hours after inoculation with *B. japonicum*. We found that GUS expression of some candidates was induced following bacterial inoculation and identified at least two distinct expression patterns in the cortex (Figure 3). After inoculation, one GUS expression pattern (*Glyma.04G244200*, *Glyma.15G012100*, and *Glyma.17G103500*) was detected in both the cortical cells and the nodule primordia, whereas another GUS expression pattern (*Glyma.12G197300*) was detected only in the surrounding cortical cells of the nodule primordia (Figure 3). *Glyma.12G197300* encodes a GRAS family transcription factor and previous studies identified two GRAS domain proteins, *NSP1* and *NSP2*, required for nodule morphogenesis (Kaló et al., 2005; Smit et al., 2005; Hossain et al., 2019). Thus, it is tempting to hypothesize that this gene plays a role in nodule primordium organogenesis.

### *GmGA3ox 1a* Regulates Nodule Development in Cortical Cells

Three gibberellin (GA) synthesis genes, *GmGA20ox a* (*Glyma.04G244200*), *GmGA20ox b* (*Glyma.06g119100*), and *GmGA3ox 1a* (*Glyma.15G012100*), were up-regulated in both TRAP-seq data sets (72 and 96 hpi) (Supplementary Table 1). We further showed that promoter-GUS expression of *GA20ox a* and *GmGA3ox 1a* was induced by *B. japonicum* inoculation in soybean root cortical cells (Figure 3). In previous studies, GA levels were found to increase in developing nodules (Ferguson and Mathesius, 2003) and GAs regulated nodule primordia establishment (Ferguson et al., 2005; Lievens et al., 2005). Moreover, both GA biosynthesis genes identified were also up-regulated in an RNA-seq data set generated using the nodulation zone of soybean roots inoculated with *B. japonicum* (Hayashi et al., 2012). Collectively these results strongly support the notion that GA levels likely play an important role in establishing nodulation.

**FIGURE 4:**
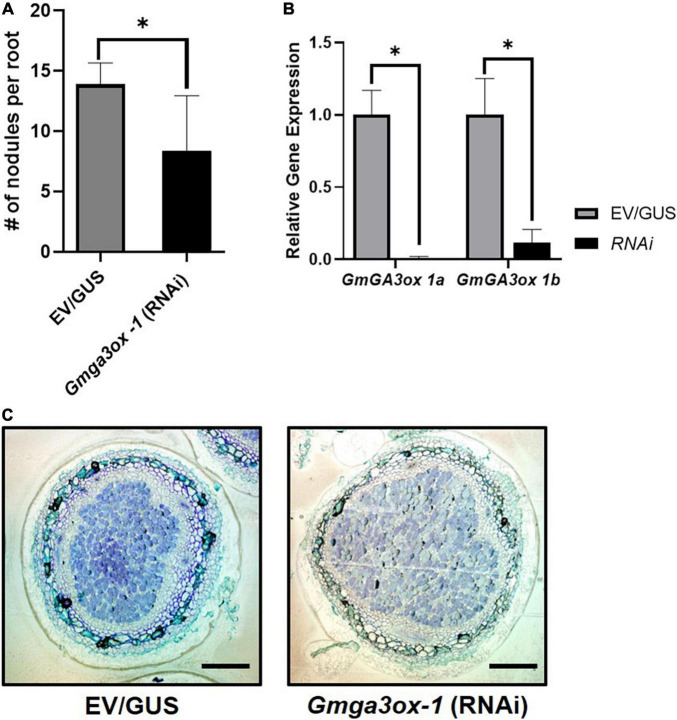
Quantification of relative nodule numbers and nodule structure in the *Gmga3ox 1*-RNAi transgenic hairy root. **(A)** Study of *Gmga3ox 1*-RNAi transgenic hairy root nodule density (Nodule number/Root fresh weight) in comparison to control vector (GUS) transgenic hairy root. **(B)** Relative gene expression of *GmGA3ox-1a* and *GmGa3ox-1b* in RNAi transgenic hairy root. **(C)** Nodule structure of *Gmga3ox-1*-RNAi transgenic hairy root after 21 days post-inoculation of *B. japonicum*. Error bar represents ± SE and * indicates *t*-test significance at *P* < 0.001 (scale bar = 200 μm).

To test whether GA synthesis genes are involved in soybean nodule primordia establishment, we used an RNAi construct to specifically silence the expression of *GmGA3ox 1a* in transgenic hairy roots. Since there are homologous *GmGa3ox 1* genes in soybean, we designed an RNAi construct that targeted both *GmGA3ox 1a* and *GmGA3ox 1b*. Knockdown of both genes led to a significant reduction in nodule density (Figure 4A). However, microscopic analysis of sectioned nodules showed that RNAi-knockdown did not visually affect nodule morphology (Figure 4C). Expression of *GmGA3ox 1a* and *GmGA3ox 1b* was significantly reduced in comparison to empty vector or GUS-RNAi controls, confirming that the reduction in nodule density was caused by gene knockdown (Figure 4B). Taken together, these results indicate that *GmGA3ox 1a* and or *GmGA3ox 1b* regulate the nodule density likely by controlling GA levels.

## Discussion

The root cortex is a critical cell layer that gives rise to the nodule primordium and is traversed by the growing infection thread during rhizobial infection. While our understanding of early epidermal responses and later nodule-specific events has greatly expanded through mutant studies and previous functional genomic analysis, our knowledge of cortex-specific events is relatively limited, largely due to the problem of effectively sampling these processes. However, a few genes have been identified as markers for cortex infection. A good example is *Enod40*, which has long served as a key nodule organogenesis marker expressed in the cortex during early nodule development (Niyikiza et al., 2020). In the current study, we specifically profiled the cortical-specific response to rhizobial inoculation using the TRAP-seq method enabled by our identification of a cortex-specific promoter. The list of differentially expressed genes identified by our analysis contributes to our understanding of the processes occurring within this tissue during the early stages of nodulation, but also represents a toolbox of genes that can be subsequently used to define these processes in greater detail.

The formation of a nitrogen-fixing nodule involves two diverse developmental processes in the legume root: infection thread formation and growth initiated in the epidermal cells and nodule primordia formation in the cortex (Ding and Oldroyd, 2009). A very large literature exists focusing on early nodulation events that define the initial interaction of rhizobia with root hair cells (Cárdenas et al., 2008; Oldroyd and Downie, 2008; Puppo et al., 2013; Hossain et al., 2015). For example, in previous work, we identified ∼2,000 genes whose expression responded to *B. japonicum* infection in soybean root hairs (Libault et al., 2010). The spatiotemporal differences in gene expression are critical to successful rhizobial infection and nodule development. For example, *NIN*, which encodes an RWP-RK-containing TF, is a master regulator of multiple genes/processes that, depending on the developmental time point, has either a positive or negative effect on nodule organogenesis, including IT formation. *NIN* initiates IT by inducing expression of legume pectate lyase *LjNPL* and nuclear transcription factor Y subunit A-1 (*MtNF-YA1*) (Yano et al., 2008; Xie et al., 2012; Laporte et al., 2014). Furthermore, *LjNF-YA1* and *LjNF-YB1* promote cortical cell division in an *LjNIN*-dependent manner (Soyano et al., 2013). When we compare our soybean cortex TRAP-seq data to the RNA-seq results derived from soybean root hair, we find, for example, expression of the *GmNIN-1a*, *GmNIN-2*, *GmNIN-2b*, and *GmNF-YA1* genes are induced in both tissues upon *B. japonicum* infection. These results are consistent with the dual function of the transcription factors *NIN* and *NF-YA1* in IT formation and cortical cell division during nodule development. Interestingly, in both root hair and cortical cells, the *GmNPL* gene responded significantly to *B. japonicum* infection. In a previous study, *DEL1*, which encodes a pectate lyase (PEL) precursor, was found to control cell division by regulating cell cycle progression in rice (Leng et al., 2017). Taken together, these findings provided insights on the possibility that the *NIN*-dependent *NPL* plays a role in nodule primordia formation by regulating cell division. The ability to compare gene expression in specific tissues, as exemplified by the comparison between the cortex TRAP-seq and root hair RNA-seq data sets, should aid in the ability to further decipher the specific epidermal and cortical programs involved in rhizobial infection and nodule development.

Nodule development is mediated by complex transcriptional networks. A number of transcriptional regulators have been discovered in various legumes in previous studies. Additionally, it is important to understand these diverse transcriptional regulatory networks in order to conduct nodule development research (Emms and Kelly, 2017; Lee et al., 2017). All plants have undergone multiple rounds of whole-genome duplication with the last soybean duplication event occurring roughly 15 mya. It has been estimated that roughly 70% of all soybean genes are duplicated and it is common to find four copies of any specific gene, which complicates functional analysis. For example, soybean has two *NSP1* and *NSP2* homologs. Interestingly, comparing our results to root hair transcriptome data reveals that these homologous genes have distinct spatiotemporal expressions (Hayashi et al., 2012; Figure 2D and Supplementary Table 3). These homologous genes were induced during the root hair infection stage, whereas only *GmNSP1a* and *GmNSP2b* were induced during early nodule development in cortical cells. Our findings suggest that *GmNSP1a* and *GmNSP2b* act on both root hair infection and nodule formation, but *GmNSP1b* and *GmNSP2a* act more specifically on root hair infection during the development of symbiotic root nodules. As a result, our high-resolution translatome data would be a useful tool for researchers studying soybean, which may have a more complicated transcriptional regulatory network due to the increased number of paralogs.

All developmental processes in plants, including nodule formation, are regulated by plant hormones (Ferguson and Mathesius, 2014). The need for a cytokinin receptor during nodule formation demonstrates that the plant hormone cytokinin is a critical component in nodule organogenesis (Tirichine et al., 2007). Abscisic acid (ABA) is typically believed to act as a negative regulator in nodule development and seems to play a role in both the epidermis and cortex (Biswas et al., 2009; Ding and Oldroyd, 2009). Other plant hormones, including positive regulators such as auxin, brassinosteroids, and GA, as well as negative regulators such as JA and ethylene, have been implicated in nodule development (Mathews et al., 1989; Ferguson and Mathesius, 2003; Ferguson et al., 2005; Sun et al., 2006; Kinkema and Gresshoff, 2008). In this research, we showed that GA biosynthesis genes (*GmGA20ox a* and *GmGA3ox 1a*) were induced by *B. japonicum* inoculation and numerous other phytohormone-related genes were identified in the MapMan sub-group analysis. Furthermore, we confirmed that *GmGA3ox* was involved in early nodule development using RNAi-transgenic hairy root system. These findings serve as an example of how our data may be further used to examine specific functions within the cortex related to nodulation and emphasize, in this specific example, the importance of hormones in these processes.

To summarize, this study demonstrates that TRAP-seq is a reproducible and powerful technique for generating valuable hypotheses that aid in analyzing the complex mechanism of soybean nodule formation.

## Materials and Methods

### Plant Materials, Conditions, and Bacterial Strains

Seeds of cultivar Williams 82 of soybean (*Glycine max*) were surface sterilized, germinated, and grown in vermiculite: perlite soil mixture (3:1) or germination paper in a growth chamber with a 16-h light and 8-h dark photoperiod, set to 23^°^C during the dark and 26^°^C during the light regime at 80% humidity. The plants were inoculated with *B. japonicum* strain USDA110. We used wild-type *B. japonicum* USDA110 for each experiment. *B. japonicum* cells were grown at 30^°^C for 3 days in HM medium (Cole and Elkan, 1973) with the appropriate antibiotics. After 3 days of culture, bacteria were pelleted, washed twice with sterilized deionized (DI) water, and diluted to an OD_600_ of 0.1 for inoculation in sterilized DI water. Plants were supplied with B&D (Broughton and Dilworth, 1971) with no nitrogen nutrient solution or with 0.5 mM NH_4_NO_3_ as in non-infected experiments. Tissues such as roots, nodules, and transgenic hairy roots were collected for statistical analysis from various experiments in this study and/or frozen immediately in liquid nitrogen. Three biological replicates were used in each experiment.

### Cloning Plasmid Construction

The promoter regions of *Glyma.18g300800* were amplified by PCR using Williams 82 genomic DNA using primers described in Supplementary Table 5 and cloned into *pDONR/Zeo* by BP reactions. Next, the plasmids (*pDONR/Zeo* constructs harboring the *Glyma.18g300800* promoter) were recombined into the *pYXT1* (Xiao et al., 2010) binary vector using the Gateway LR Clonase II enzyme mixes (Invitrogen).

For the target gene RNAi constructs, ∼195 bp of *Glyma.15G12100* (*GmGa3ox 1a*) gene specific fragment was amplified using primers described in Supplementary Table 5. Amplified fragments were cloned into the pDONR/Zeo vector. The resulting positive plasmid was recombined into the *pCAMGFP-CvMV-GWi* binary vector (Graham et al., 2007) using the Gateway LR Clonase II enzyme mixes.

For the promoter:GUS constructs, the promoter regions of *Glyma.15G12100*, *Glyma.12G197300*, *Glyma.04G244200*, and *Glyma.17G03500* were amplified by PCR using gDNA using primers described in Supplementary Table 5 and cloned into *pSoyGUS* binary vector (Hossain et al., 2019) using Gibson assembly enzyme (NEB).

### Soybean Hairy Root Transformation and Microscopy

To express the target genes in soybean and to investigate their localization, the appropriate plasmid constructs were transformed into *Agrobacterium rhizogenes K599* and used for hairy root transformation as described (Song et al., 2021). Transgenic hairy roots were selected based on constitutive GFP expression using dissecting fluorescence microscopy before and after inoculation with *B. japonicum*. Nodule numbers were counted in each root 4 weeks after *B. japonicum* inoculation. Harvested transgenic tissues (root and nodule) were frozen in liquid nitrogen and used for subsequent downstream studies. The nodules were fixed in 4% paraformaldehyde in sodium phosphate buffer and embedded in paraplast to clearer understanding and study their morphology and structure. After staining the nodule section with toluidine blue (0.1%), it was washed with water and then dried at room temperature prior to microscopic analysis. Three independent experiments were conducted for the statistical study, and each experiment setup included at least 15 plants per replicate for quantification of nodule development. For statistical analysis, the student’s *t*-test was used.

### Translating Ribosome Affinity Purification Followed by RNA Sequencing (TRAP-Seq)

TRAP was modified based on the method by Zanetti et al. (2005) as described previously (Castro-Guerrero et al., 2016). The optimized protocol required 10 g of root material per sample when using cortex-layer specific HisFLAG-GmRPL18 expressing hairy root. This was sufficient to obtain at least 500 ng of mRNA for library preparation and subsequent RNA sequencing. In this study, three biological replicates were prepared for each time point (72 and 96 h) for each treatment (mock- and *B. japonicum*-inoculated).

### Preparation and Sequencing of RNA-Sequencing Libraries

Extracted RNAs from TRAP were shipped frozen on dry ice to the University of Missouri’s DNA Core Facility for preparation of an RNA-Seq library using the TruSeq stranded mRNA Kit (Illumina, San Diego, California, United States) and high-throughput sequencing (75 bp paired-end) on an Illumina NextSeq 500.

### Analysis of Translating Ribosome Affinity Purification RNA Sequences

For all the raw reads FASTQ files, we firstly checked their quality with FastQC (Wingett and Andrews, 2018), which provides a quick view on the quality of all the raw sequence reads from multiple analyses, ranging from the sequence quality, GC content, to library complexity, and produce a report in the HTML format, and we filtered reads based on the quality scores. Then high-quality reads were aligned to the soybean reference genome (*Glycine max* Wm82.a4.v1) by STAR (Dobin et al., 2013), and we merged all the results into one big raw count expression matrix. edgeR (Robinson et al., 2009) was used to identify genes with differential expression abundance between 72 and 96 hpi TRAP RNA samples. Heat map was generated by per gene z-score that was computed from log2 fold-change values. All RNA-seq data are deposited at NCBI (GEO accession: GSE192785).

### Histochemical GUS Staining

GUS staining was performed as described previously (Jefferson et al., 1987). Briefly, transgenic hairy roots were stained overnight at 37°C in 10 ml staining solution (1 mg ml^−1^ 5-bromo-4-chloro-3-indolyl-β-d-glucuronide, 100 mM sodium phosphate, pH 7.0, 1 mM EDTA, 0.05% Trition X-100, 5 mM potassium ferrocyanide, 5 mM potassium ferricyanide). Images were collected using bright field on a Leica DM5500B upright microscope with color camera.

### Quantitative Real-Time RT-PCR

To measure the steady-state level of transcripts, total RNA was extracted from transgenic hairy root samples and treated with RNase-free DNase I. Two micrograms of total RNA were used for the reverse transcription using M-MLV Reverse Transcriptase (Promega), and 1 μl of resulting first-strand cDNA (20 μl) was used as a PCR template for the quantitative real-time RT-PCR. Quantitative PCR analysis was performed using SYBR Green PCR mix (ABI enzyme) with a CFX96 PCR machine (Bio-Rad). *Cons4* (Libault et al., 2008) was used as an internal reference gene to normalize the relative level of each transcript. Primers used in this experiment were described in Supplementary Table 5.

### Immunoprecipitation and Western Blot Analysis

Total proteins were extracted from transgenic hairy roots. For western blot analyses, total proteins were extracted from approximately 200 mg of tissue. The following antibodies were used in these experiments: monoclonal anti-FLAG-HRP (Sigma, 1/3,000). One gram of seedlings or transiently expressed hairy root was used for the immunoprecipitation experiments. The tissue was ground in liquid nitrogen, re-suspended in 1 ml of immunoprecipitation buffer (50 mM Tris pH 7.5, 150 mM NaCl, 1% Triton X-100 and protease inhibitor cocktail) and left on a rotating wheel for 30 min at 4^°^C. Samples were then centrifuged for 10 min at 20,000 × g at 4^°^C. Immunoprecipitations were carried out on 1 mg of total proteins using the EZview™ Red ANTI-FLAG ^®^ M2 Affinity Gel (Sigma) according to the manufacturer’s protocol.

## Data Availability Statement

The original contributions presented in the study are publicly available. This data can be found here: NCBI GEO, GSE192785.

## Author Contributions

GS and JS designed the experiments. JS, MT-S, BM-L, YC, and LS performed the research and analyzed the data. BM-L and DX revised the manuscripts. JS wrote the manuscript with the input from GS. All authors contributed to the article and approved the submitted version.

## Conflict of Interest

The authors declare that the research was conducted in the absence of any commercial or financial relationships that could be construed as a potential conflict of interest.

## Publisher’s Note

All claims expressed in this article are solely those of the authors and do not necessarily represent those of their affiliated organizations, or those of the publisher, the editors and the reviewers. Any product that may be evaluated in this article, or claim that may be made by its manufacturer, is not guaranteed or endorsed by the publisher.

## Footnotes

1
https://phytozome-next.jgi.doe.gov/info/Gmax_Wm82_a4_v1

2
https://www.plabipd.de/portal/web/guest/home1

## Supplementary Material

Supplementary Figure 1 Functional classification and comparison of TRAP-seq DEGs. **(A)** Biological groups of DEGs categorized according to sequence similarity by MapMan. Functional subcategories of **(B)** transcription factors and **(C)** phytohormones comparing between 72 and 96 hpi DEGs. Blue and green bars represent the number of specific and common genes for each time point, respectively.

Supplementary Table 1 Fold change of DEGs in TRAP-seq.

Supplementary Table 2 Comparison of selected gene expression in TRAP-seq and previous RNA-seq data.

Supplementary Table 3 Comparison of nodulation gene expression in TRAP-seq and previous RNA-seq data.

Supplementary Table 4 Functional annotation of TRAP-seq using Mercator4.

Supplementary Table 5 Primer used for this study.
