# Supplementary figures and images for: High-Resolution Translatome Analysis Reveals Cortical Cell Programs During Early Soybean Nodulation
Source: Front Plant Sci. 2022 Apr 14;13:820348. doi: 10.3389/fpls.2022.820348 (PMC9048599; doi:10.3389/fpls.2022.820348)

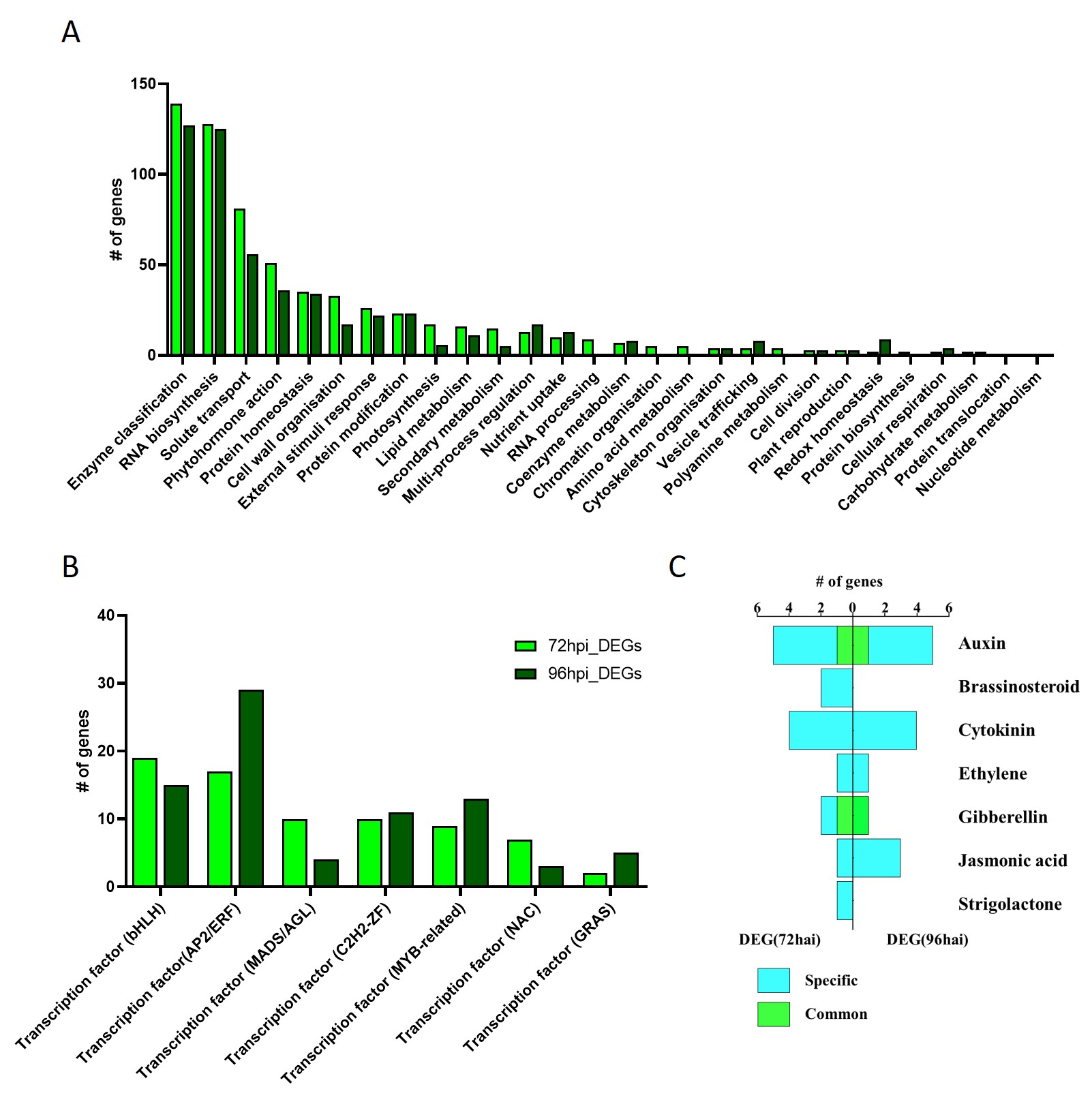

Supplement: Supplementary Figure 1 — Functional classification and comparison of TRAP-seq DEGs. (A) Biological groups of DEGs categorized according to sequence similarity by MapMan. Functional subcategories of (B) transcription factors and (C) phytohormones comparing between 72 and 96 hpi DEGs. Blue and green bars represent the number of specific and common genes for each time point, respectively. [file Image_1.jpeg]
